# Supplementary material for: Peripartum Predictors of the Risk of Postpartum Depressive Disorder: Results of a Case-Control Study
Source: Int J Environ Res Public Health. 2020 Nov 24;17(23):8726. doi: 10.3390/ijerph17238726 (PMC7727836; doi:10.3390/ijerph17238726)
Supplement: Supplementary file 1 [file ijerph-17-08726-s001.pdf]

Edinburgh Perinatal/Postnatal Depression Scale (EPDS)

For use between **28–32 weeks** in **all** pregnancies and **6–8 weeks** postpartum

Name: ______________________ Date: ______________ Gestation in Weeks: ______

As you are having a baby, we would like to know how you are feeling. Please mark "X" in the box next to the answer which comes closest to how you have felt in the **past 7 days**–not just how you feel today.

In the past 7 days:

1. I have been able to laugh and see the funny side of things
   - 0 ☐ As much as I always could
   - 1 ☐ Not quite so much now
   - 2 ☐ Definitely not so much now
   - 3 ☐ Not at all

2. I have looked forward with enjoyment to things
   - 0 ☐ As much as I ever did
   - 1 ☐ Rather less than I used to
   - 2 ☐ Definitely less than I used to
   - 3 ☐ Hardly at all

3. I have blamed myself unnecessarily when things went wrong
   - 3 ☐ Yes, most of the time
   - 2 ☐ Yes, some of the time
   - 1 ☐ Not very often
   - 0 ☐ No, never

4. I have been anxious or worried for no good reason
   - 0 ☐ No, not at all
   - 1 ☐ Hardly ever
   - 2 ☐ Yes, sometimes
   - 3 ☐ Yes, very often

5. I have felt scared or panicky for no very good reason
   - 3 ☐ Yes, quite a lot
   - 2 ☐ Yes, sometimes
   - 1 ☐ No, not much
   - 0 ☐ No, not at all

6. Things have been getting on top of me
   - 3 ☐ Yes, most of the time I haven't been able to cope
   - 2 ☐ Yes, sometimes I haven't been coping as well as usual
   - 1 ☐ No, most of the time I have coped quite well
   - 0 ☐ No, I have been coping as well as ever

7. I have been so unhappy that I have had difficulty sleeping
   - 3 ☐ Yes, most of the time
   - 2 ☐ Yes, sometimes
   - 1 ☐ Not very often
   - 0 ☐ No, not at all

8. I have felt sad or miserable
   - 3 ☐ Yes, most of the time
   - 2 ☐ Yes, quite often
   - 1 ☐ Not very often
   - 0 ☐ No, not at all

9. I have been so unhappy that I have been crying
   - 3 ☐ Yes, most of the time
   - 2 ☐ Yes, quite often
   - 1 ☐ Only occasionally
   - 0 ☐ No, never

10. The thought of harming myself has occurred to me
    - 3 ☐ Yes, quite often
    - 2 ☐ Sometimes
    - 1 ☐ Hardly ever
    - 0 ☐ Never

Total Score ☐

Talk about your answers to the above questions with your health care provider.

Translations for care-provider use available on PSBC website: perinatalservicesbc.ca.

The Royal College of Psychiatrists 1987. From Cox, JL, Holden, JM, Sagovsky, R (1987). Detection of postnatal depression. Development of the 10-item Edinburgh Postnatal Depression Scale. British Journal of Psychiatry. 150, 782–786. Reprinted with permission.
